# Efficacy and Safety of Intravitreal Conbercept, Ranibizumab, and Triamcinolone on 23-Gauge Vitrectomy for Patients with Proliferative Diabetic Retinopathy

**DOI:** 10.1155/2018/4927259

**Published:** 2018-06-25

**Authors:** Jinglin Cui, Hong Chen, Hang Lu, Fangtian Dong, Dongmei Wei, Yan Jiao, Steve Charles, Weikuan Gu, Lin Wang

**Affiliations:** ^1^Center of Integrative Research, The First Hospital of Qiqihar City, Qiqihar, Heilongjiang 161005, China; ^2^Department of Orthopedic Surgery and BME-Campbell Clinic, University of Tennessee Health Science Center, Memphis, TN 38163, USA; ^3^Department of Ophthalmology, Peking Union Medical College Hospital, Beijing 100730, China; ^4^Department of Ophthalmology, University of Tennessee Health Science Center, Memphis, TN 38163, USA; ^5^Charles Retina Institute, Germantown, TN 38138, USA; ^6^Research Service, Veterans Affairs Medical Center, 1030 Jefferson Avenue, Memphis, TN 38104, USA

## Abstract

**Introduction:**

To compare the effect and safety of intravitreal conbercept (IVC), intravitreal ranibizumab (IVR), or intravitreal triamcinolone acetonide (IVTA) injection on 23-gauge (23-G) pars plana vitrectomy (PPV) for proliferative diabetic retinopathy (PDR).

**Methods:**

Fifty patients (60 eyes) of varying degrees of PDR were randomly grouped into 3 groups (1 : 1 : 1) (*n* = 20 in each group). The 23-G PPV was performed with intravitreal conbercept or ranibizumab injection 3–7 days before surgery or intravitreal TA injection during surgery. The experiment was randomized controlled, with a noninferiority limit of five letters. Main outcome measures included BCVA, operation time, incidence of iatrogenic retinal breaks, endodiathermy rate, and silicone oil tamponade.

**Results:**

At 6 months after surgery, there were no significant differences of BCVA improvements, operation time, incidence of iatrogenic retinal breaks, endodiathermy rate, silicone oil tamponade, vitreous clear-up time, and the incidence of intraoperative bleeding between the IVC and IVR groups (all *P* values ≥ 0.05), but they were significantly different from the IVTA group (all *P* values < 0.05). IOP increases did not show significant differences between the IVC and IVR groups, but both were significantly different with the IVTA group. More patients had higher postoperative IOP in the IVTA group.

**Conclusions:**

The intravitreal injection of conbercept, ranibizumab, or TA for PDR had a significant different effect on outcomes of 23-G PPV surgery. Conbercept and ranibizumab can reduce difficulty of the operation, improve the success rate of PPV surgery, and decrease the incidence of postoperative complications.

## 1. Introduction

Proliferative diabetic retinopathy (PDR) is the leading cause of blindness among DR in diabetic patients [1–6]. PDR can lead to vitreous hemorrhage, traction detachment from fibrous proliferation, or neovascular glaucoma [7]. The current standard treatment for PDR is panretinal photocoagulation (PRP), combined with PPV whenever necessary. However, PRP is naturally destructive and has several potential adverse effects on visual function, including constriction of the peripheral visual field and reductions in night vision, contrast sensitivity, and color perception. Furthermore, it has been known that in the absence of intravitreal administration of ranibizumab or triamcinolone acetonide (TA), PRP can negatively affect vision and macular thickness in patients with diabetic macular edema (DME) [8]. In the surgery of advanced PDR, the occurrence of intraoperative hemorrhage when dissecting epiretinal neovascular membrane will seriously affect visualization of the surgical field. In addition, repeated bleeding can prolong the operation time, increase the frequency of instrument exchange, and greatly increase the occurring rate of complications [9].

In order to reduce the chance of complications, a variety of drugs have been utilized in PPV for PDR. TA (Kenalog, Bristol-Myers Squibb Company, Princeton, NJ) (Kenakolt-A, Bristol Pharmaceuticals KK, Tokyo, Japan) is a water-insoluble steroid that inhibits various inflammatory reactions. It has been confirmed that it can aid visualization of transparent vitreous, reduce the degree of postoperative inflammation, and decrease the incidence of reoperation owing to epiretinal membrane formation in TA-assisted PPV for PDR [10–12]. In recent years, the important role of excessive release of vascular endothelial growth factor (VEGF) in many retinal vascular diseases has been unanimously recognized worldwide, including in PDR surgery [13–15]. Ranibizumab (Lucentis; Genentech Inc., South San Francisco, CA) and bevacizumab (Avastin; Genentech Inc., South San Francisco, CA) are monoclonal antibodies, militating by block VEGF-A. Studies showed that both of them can result superior visual acuity and central retinal thickness, reduce the duration of surgery, achieve fewer retinal breaks, and lessen intraoperative bleeding and also lead fewer endodiathermy applications [16]. However, bevacizumab has not been approved for use in intraocular injections in China. Conbercept (Langmu; Kanghong Inc., Sichuan, China) is a VEGF receptor (VEGFR) fusion protein. In late 2013, it received the new drug certificate, drug registration approval, and GMP certification from State Food and Drug Administration in China and has been widely used, accompanied by neovascularization vitreoretinopathy, such as neovascular age-related macular degeneration (AMD). It functions by competitively inhibiting the binding of VEGF with its receptor by blocking multiple targets, VEGF-A, VEGF-B, and placental insulin-like growth factor (PlGF) [17]. Most recently, conbercept has been reported to be an effective adjunct for the intravitreal conbercept (IVC) injection before vitrectomy for proliferative diabetic retinopathy (PDR) [18]. Thus, TA has traditionally been used PPV for PDR. Conbercept has been recently tested for its benefit when it was used PPV for PDR, mostly in Europe. Conbercept has been mostly tested in China. These three of them have never been directly compared. This study aims to compare the efficacy and safety of PPV when assisted by conbercept, ranibizumab, and TA intravitreal injection for PDR.

## 2. Methods

### 2.1. Study Population

This study adheres to the guidelines of the Declaration of Helsinki. The study was approved by the Institutional Review Board of the First Hospital of Qiqihar City. The protocol number is 2006-04. Patients' consents were given to all participants, and all patients signed the consents before participating the study. Between Jan 2015 and Dec 2015, 60 eyes from 53 patients were collected of varying degrees of PDR in the First Hospital of Qiqihar. There were 33 (55%) male and 27 (45%) female. The age was between 29 and 78 years old, with the average age of 58.83 (±3.62). Mean duration of DM was 26.57 ± 5.82 years. All patients had a history of DM, with 14 (23.3%) cases of type 1 DM and 46 (76.7%) cases of type 2 DM. Visual acuity was tested using Early Treatment Diabetic Retinopathy Study (ETDRS) charts at 4 m [18]. The BCVA was from HM to 20/80 as determined by protocol trial lens refraction. Other examinations included slit lamp directly, indirect ophthalmoscopy, IOP measurement, B-scan ultrasonography, fundus fluorescein angiography (FFA), and optical coherence tomography (OCT). Patients were selected for the PPV treatments (Table 1) based on the existence of extent of vitreous hemorrhage, retinal proliferation or traction retinal detachment, and other serious PDR. Exclusion criteria included those who received prior intravitreal injection, underwent vitreous or retinal surgeries, and glaucoma. Patients with abnormal blood coagulation indexes and other diseases of surgical contraindication were also excluded [19]. Before treatment, patients were provided with informed consent, the risks of surgery, and intraocular injection, and surgical complications related to the treatments were discussed. All patients understood the content and signed the informed consent. The study was approved by the First Hospital of Qiqihar Committees for Medical and Health Research.

### 2.2. Study Procedures

Patients were randomly divided into IVC, IVR, and IVTA groups (1 : 1 : 1) (*n* = 20 eyes in each group). Mean BCVA was 27.83 ± 6.78, 25.31 ± 4.23, and 28.46 ± 7.55 (ETDRS letters) in the IVC, IVR, and IVTA groups, respectively. The IVC group were 20 eyes in 17 patients, including 11 eyes (9 cases, 55%) of male and 9 eyes (8 cases, 45%) of female. Patients received 0.5 mg (0.05 ml, 10 mg/ml) intravitreal injections of conbercept [20] while the IVR group were 20 eyes of 20 patients, including 14 eyes (14 cases, 70%) of male and 6 eyes (6 cases, 30%) of female. Patients received 0.5 mg (0.05 ml, 10 mg/ml) intravitreal injections of ranibizumab [21]. PPV in both IVC and IVR groups was completed within 3–7 days after injection, and TA was not used during the surgery in both groups. The IVTA group were 20 eyes in 16 patients, including 12 eyes (11 cases, 60%) of male and 8 eyes (5 cases, 40%) of female. Patients received 4 mg (0.5 ml, 8 mg/ml) intravitreal injections of TA during the PPV [22]. The TA in the group of IVTA was removed during the surgery, with no remaining in the vitreum at the end of surgery. Three drugs were acquired commercially, and batch numbers for all vials used in the study were registered. Sterile techniques were used for every injection. Ophthalmic antibiotics and prophylactic peri-intravitreal injection were not used. Topical anesthetics were used (0.4% oxybuprocaine hydrochloride eye drops, Santen Pharmaceutical Co. Ltd.). The periocular skin, eyelids, and eyelashes were disinfected with 10% povidone-iodine swabs, and 5% povidone-iodine ophthalmic solution was applied to the ocular surface. All the patients received 23-G (Gauge) PPV (Alcon). The surgeries were performed by two experienced vitreoretinal specialist (Fangtian Dong and Hang Lu), who were masked from the patient information. The choice of tamponade was made between C3F8 gas or silicone oil depending on the difficulty and complexity of the surgery, such as the severity of traction, size and number of retinal breaks or detachment, presence of iatrogenic breaks, retinectomy, severe bleeding, and other intraoperative complications [21]. Intraoperative panretinal endolaser photocoagulation was used, whenever necessary, at the end of the PPV surgery [23]. Ophthalmic antibiotics (5% levofloxacin eye drops, Santen, Japan) were used from the first day after surgery for 3 days, 4 times/d. Follow-up time was 6 months.

### 2.3. Data and Statistical Analysis

The primary outcomes were mean BCVA (ETDRS chart) monthly, operation time, incidence of iatrogenic retinal breaks, endodiathermy rate, and silicone oil tamponade. Secondary outcomes included average vitreous clearing time and the frequency of intraoperative and postoperative bleeding, PRP completion rate, reoperation probability, and intraocular pressure (IOP) in each group. Vitreous clearing time was defined as the interval between the end of surgery and the time at which the vitreous cleared up completely. Increased IOP was defined as an intraocular pressure > 21, which occurred within 24 hours after injections. To prevent effect of silicone oil on postoperative visual acuity, the final results of BCVA were determined after silicone oil removal. For patients with cataract after surgery, BCVA was measured after cataract extraction combined with intraocular lens implantation. Complications of cataract surgery were not included in this study.

The margin of clinical noninferiority was defined as five letters on the ETDRS visual acuity chart. Statistical analysis of the primary outcome variable, the mean change in BCVA from baseline to 6 m follow-up, was performed on data from the per protocol population (patients attending the 6 m visits). The mean scores of the primary outcome variables in three treatment groups were compared to each other using the independent samples *t*-test. The same statistical procedure was applied when analyzing the data according to the intent-to-treat principle, using multiple imputing to replace missing observations at 6 m follow-up.

Statistical analysis of secondary outcomes was performed only on data from the per protocol population, the operation time by independent samples *t*-test; if *p* < 0.05, the difference was considered statistically significant.

## 3. Results

### 3.1. Patients and Treatments

**Figure 1:**
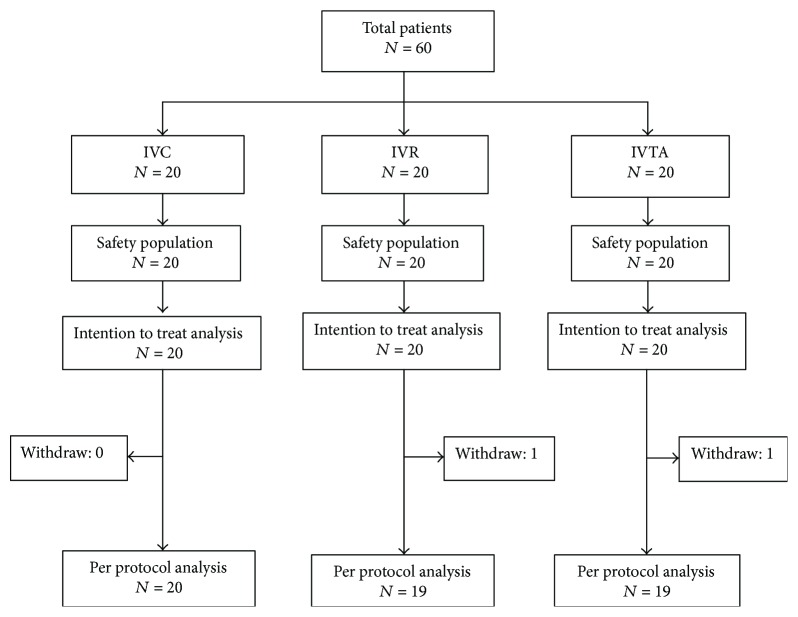
Study flow chart.

60 patients were included in the treatment and safety analysis. The 6-month visits were completed by 58 (96.7%) patients (Supplementary Table 1). Two (3.3%) patients were lost to follow-up (one was in the IVR group and the other in the IVTA group). The primary analysis followed the intent-to-treat principle and included all randomized eyes (Figure 1). There were no substantial differences among the groups regarding age, sex, IOP, BCVA, and DR degree of severity in baseline characteristics (Table 1). To obtain 3 homogeneous groups of surgical complexity, we assigned scores from 0 to 3 for the following preoperative parameters: (1) vitreous hemorrhage (VH), (2) previous retinal laser photocoagulation, and (3) morphological types of retinal detachment, such as hammock, central diffuse, and table-top [24]. There was no significant difference in these scores. All patients did not receive PPV or intravitreal injection treatment, but some of them have received PRP treatment (cases were 4, 2, and 2 in 3 groups, resp.) (Table 2). The means and standard deviation of three groups showed that there was no difference among them.

### 3.2. Primary Outcomes

**Figure 2:**
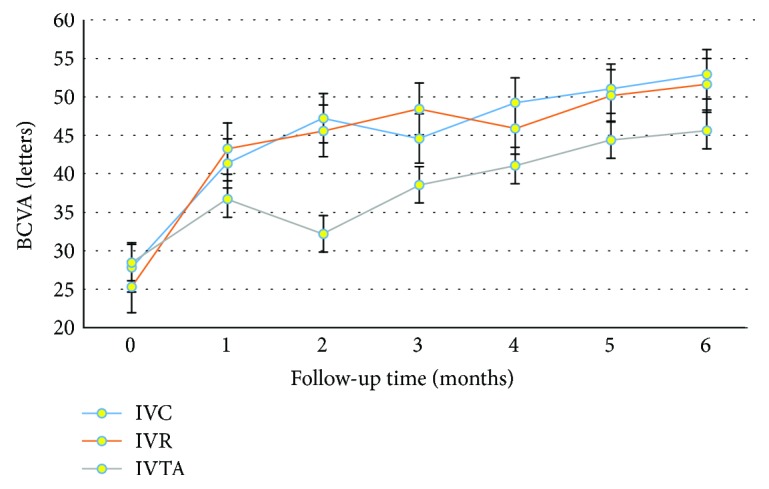
The mean changes in BCVA from baseline in IVC, IVR, and IVTA groups over 6 m were as indicated by the ETDRS chart letters. BCVA gradually increased after treatments in all three groups. The increases of BCVA were the most at the end of the first month. At the end of 6 m, the mean BCVA was improved by 25.10 ± 3.73, 26.32 ± 4.06, and 17.16 ± 2.87 letters in IVC, IVR, and IVTA groups, respectively (all *P* values < 0.05).

At the end of 6 m follow-up, the mean improvements in the IVC, IVR, and IVTA groups, respectively, were as follows (Figure 2): BCVA (ETDRS charts) was 25.10 ± 3.73, 26.32 ± 4.06, and 17.16 ± 2.87; the mean operation time was 56.65 ± 6.52, 54.89 ± 6.46, and 77.32 ± 6.36; the incidence of iatrogenic retinal breaks was 2 (10.0%), 2 (10.5%), and 8 (42.1%) cases; the endodiathermy rate was 5 (25.0%), 6 (31.6%), and 12 (63.2%) cases; and silicone oil tamponade was 9 (45.0%), 9 (47.4%), and 15 (78.9%) cases. There were no significant differences in BCVA improvements, operation time, incidence of iatrogenic retinal breaks, endodiathermy rate, and silicone oil tamponade between the IVC and IVR groups (all *P* values ≥ 0.05). However, each of these two groups showed significant difference with the IVTA group (all *P* values < 0.05) (Table 3).

### 3.3. Secondary Outcomes

**Figure 3:**
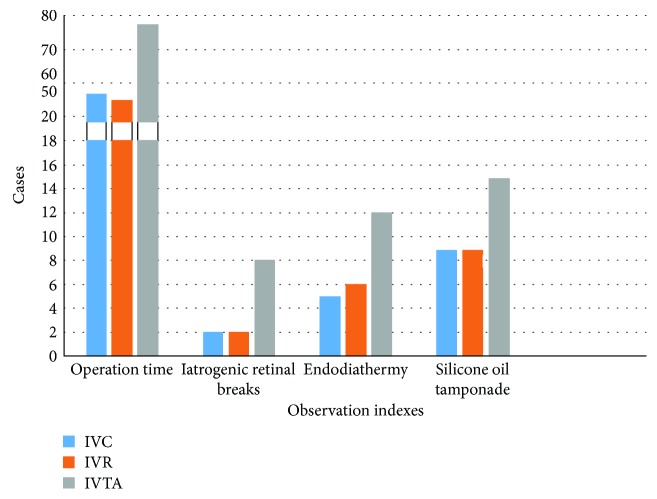
Comparison of outcomes of IVC, IVR, and IVTA groups at 6 m. There were no significant differences in operation time, incidence of iatrogenic retinal breaks, endodiathermy rate, and silicone oil tamponade between IVC and IVR groups. However, each of these two groups showed significant difference with the IVTA group.

The average vitreous clear-up time was 6.10 ± 1.52, 6.32 ± 1.57, and 11.11 ± 2.38 in the IVC, IVR, and IVTA groups, respectively (Figure 3); the incidence of intraoperative bleeding was 2 (10.0%), 3 (15.8%), and 9 (47.4%) cases in the three groups, respectively; postoperative bleeding was 1 (5.0%), 1 (5.3%), and 3 (15.8%) cases in the three groups, which occurred at 5 d, 1 w, and 1.5 m, respectively. Three patients required reoperation. Two cases were treated with Chinese drugs (He Xue Ming Mu Pian and Hong Hua Huang Se Su). BCVA was measured at 3 months after treatments. PRP completion rate was 11 (55.0%), 10 (52.6%), and 6 (31.6%) cases in the IVC, IVR, and IVTA groups, respectively. Four patients needed reoperation with the distribution of 1 (5.0%), 1 (5.3%), and 2 (10.5%) in the three groups, respectively. Three of them were caused by postoperative bleeding, and 1 was caused by silicone oil emulsified into the anterior chamber. There were no significant differences in vitreous clear-up time and the incidence of intraoperative bleeding between the IVC and IVR groups, while both of these groups were significantly different from the IVTA group. However, there were no significant differences in the incidence of postoperative bleeding, PRP completion rate, and reoperation probability among the 3 groups (Table 4).

### 3.4. Adverse Events

**Figure 4:**
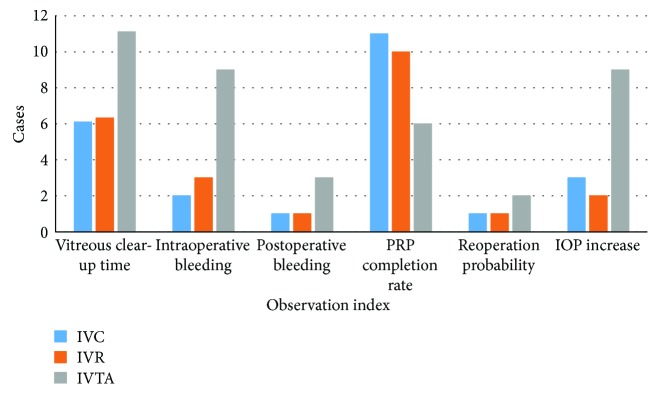
Secondary outcomes and adverse events of the IVC, IVR, and IVTA groups at 6 m. There were no significant differences in vitreous clear-up time and the incidence of intraoperative bleeding between IVC and IVR groups, while both of these groups were significantly different from IVTA group. More patients were at high IOP level in the IVTA group than the other two groups after surgeries. However, there were no statistically significant differences in the incidence of postoperative bleeding, PRP completion rate, and reoperation probability among 3 groups.

IOP increase is defined as an intraocular pressure > 25 mmHg, which appeared within 24 hours after injections. If IOP increased, subjects were monitored until intraocular pressure at 25 mmHg or less. The cases with increased IOP were 3 (15.0%), 2 (10.5%), and 9 (47.4%) in the IVC, IVR, and IVTA groups, respectively (Figure 4). There were no significant differences of IOP rate between the IVC and IVR groups, but both groups were significantly less than that in the IVTA group. Thus, more patients are at high IOP level in the IVTA group than the other two groups after surgeries. Among IOP patients, 5 were given anterior chamber tap, while others were treated with IOP-lowering drugs. The IOP of all of these patients decreased to normal ranges within 2 weeks. There were no significant differences in hypertension, cardiovascular, and cerebral vascular diseases among the 3 groups, compared with baselines (Table 5). No endophthalmitis, iris neovascularization, or TRD progression were observed during the follow-up period.

## 4. Discussion

PDR usually is extremely complicated with intraocular hemorrhage and TRD. Because of the existence of hemorrhage, exudation, and proliferation membrane during surgery in severe PDR, structures of retina are not easily identified and surgical difficulty and complexity are increased. Several studies have confirmed that VEGF plays a very important role in complex PDR [25, 26]. Due to long-term hypoxia in the occurrence and development of PDR, secretion of VEGF by retinal cells is increased, which causes new vessel hyperplasia, vitreous hemorrhage, and fibrovascular membranes and eventually leading to the TRD and severe damage to vision or even blindness [27, 28]. Clinical trials concluded that preoperative intravitreal injection of anti-VEGF drugs can reduce the intravitreal VEGF level, inhibit the activity of VEGF partially, and decrease retinal vascular leakage and neovascularization [29, 30]. Anti-VEGF drugs can also reduce the incidence of bleeding and iatrogenic holes during epiretinal membrane dissection [31, 32]. The VEGF family consists of VEGF-A, VEGF-B, VEGFC, VEGF-D, and placental growth factor (PIGF), which are related to receptors VEGFR-1, VEGFR-2, and VEGFR-3. VEGF-A can activate both VEGFR-1 and VEGFR-2. Meanwhile, VEGF-B and PIGF only bind to VEGFR-1. Also, VEGF-C and VEGF-D only bind to VEGFR-3 [33]. However, the monoclonal antibodies such as ranibizumab and bevacizumab had been found to bind VEGF-A only and lasted for only a short time [34]. Conbercept is a humanized soluble VEGFR protein which comprises extracellular domain 2 of VEGFR-1 and extracellular domains 3 and 4 of VEGFR-2, all of which are combined with the Fc region of human immunoglobulin G1 simultaneously. Based on its structure, it is predicted that it inhibits the binding of multiple VEGF receptors. Previous studies have demonstrated that extracellular domain 4 of VEGFR-2 can enhance the three-dimensional structure and efficiently advance dimerization [35]. Therefore, it is relatively stable and long lasting, in comparison with that of monoclonal antibodies. Also, preclinical studies have presented higher affinity of conbercept for VEGF than bevacizumab [36].

In addition, postoperative inflammation is also one of the major causes of postoperative complications, such as proliferative vitreoretinopathy (PVR). The postoperative inflammatory cells can secrete varieties of chemical mediators and cytokines, which stimulate the invasion of secondary inflammatory cells into the vitreoretinal tissue and activate the retinal glial cells and retinal pigment epithelium cells. These activated cells cause the proliferation of themselves, produce extracellular matrix, and contract the epiretinal membrane, thus leading to a secondary retinal detachment [37, 38]. Therefore, a reduction of postoperative inflammation is a logical strategy to prevent postoperative complications.

IVC, IVR, and IVTA are three commonly used procedures to improve the PDR operation in China. Only a single injection of TA was used early. Currently, anti-VEGF drugs in this study have been used in conjunction with PPV for PDR in China. Early studies showed that intravitreal injection of TA successfully inhibited experimental PVR in the rabbit and optic disk neovascularization in the pig [39, 40]. In the study by Enaida et al., 62 Patients with PVR, diabetic macular edema (DME), PDR, rhegmatogenous retinal detachment (RRD), and macular hole retinal detachment (MHRD) were treated with TA-assisted PPV surgeries. Results showed that 49% of patients had improved vision and a lower incidence of reoperation caused by preretinal fibrous membrane formation [41]. Also, a study showed that performing intravitreal TA injection during PPV can increase the intraoperative visualization of vitreous; therefore, it may facilitate both removal of epiretinal membrane and separation of vitreous, especially in patients with undetached vitreous [42]. TA also was confirmed sufficient to reduce postoperative inflammation, as TA particles were left on the retinal surface for a few days [43]. Ranibizumab is a humanized monoclonal antibody fragment, which lacks an Fc domain, that functions by blocking all VEGF-A isoforms [44]. Conbercept is a different VEGFR fusion protein with multiple binding targets [45]. Large randomized controlled trials (RCTs) have authenticated principally the role of anti-VEGF agents in age-related macular degeneration, retinal vascular occlusion, and diabetic macular edema [46–49]. Studies in recent years have explored the role of anti-VEGF agents in PDR either as stand-alone therapy or as an adjunct to laser or PPV. Meta-analysis suggests that the addition of IVR to PRP results in improved structural and functional outcomes at 3 months/16 weeks and supports the assertion that application of intravitreal anti-VEGF therapy before PPV has the effect of reducing operating times, increasing the ease of surgery [50]. These facts support the use of anti-VEGF agents as adjunctive therapy in patients requiring PRP or vitrectomy for complicated PDR.

In our study, 60 eyes of PDR which combined with vitreous hemorrhage in different degrees and TRD were selected. Patients were randomly divided into three groups, ignoring the severity of the disease. The results showed that the preoperative application of intravitreal injections of conbercept and ranibizumab had equal effect in improvement of visual acuity, operation time, incidence of iatrogenic retinal breaks, endodiathermy rate, frequency of silicone oil tamponade, vitreous clearing time, and the incidence of intraoperative bleeding. Compared with the IVTA group, the IVC and IVR groups had more visual acuity gains after surgeries and increased operation safeties. In PPV surgery of the IVC and IVR groups, the fibrous proliferative membranes were easily separated from the retina with a few individual of bleeding. The advantages of the IVC and IVR groups are time saving for operations and reduced risks of surgical complications.

However, the posterior hyaloid can be clearly seen after the injection of TA suspension that enhanced visualization of vitreous in the IVTA group. Nevertheless, considering the potential increased risk of glaucoma and cataract associated with the use of intravitreal corticosteroids, the use of intravitreal corticosteroid preparations to reduce the likelihood of retinopathy worsening does not seem warranted [7].

Our data indicated that there were no significant differences among the three groups in the incidence of postoperative bleeding, PRP completion rate, and reoperation probability. Thus, although IVC, IVR, and IVTA may function in variable degrees, they all improved postoperative conditions and reduced complication occurrence of PPV. Conbercept, ranibizumab, and TA also improved the completion rate of postoperative PRP, prevented the development of DR, and greatly improved the patient's prognosis. The number of eyes with IOP increase was more in the IVTA group than the other two groups, suggesting that although TA was believed able to be removed from vitreous after PPV [40], its effect on IOP continually exists to a certain extent. There were no significant differences in other adverse events, such as hypertension, cardiovascular, and cerebral vascular diseases among the 3 groups compared with baselines, suggesting that there is very little or no influence on the system events from intravitreal injections of these three drugs. The early postoperative bleeding usually was relevant to the dissection of fibrovascular membranes in surgery which occurred typically within 1 week of surgery [51]. Pretreatment with conbercept surely facilitated the reducing of postoperative bleeding early after surgery due to the regression of neovascularization, cessation of hemorrhage from all potential bleeding sources, and reintegration of retinal vascular tissue. However, due to the short-time effect of anti-VEGF drugs injected before surgery, it did not affect late VH incidence [20]. Thus, due to the short duration of time of the anti-VEGF drug pretreatment in the eye, there were no significant differences in the incidence of postoperative bleeding among the three groups.

It has been controversial on the optimal timing of preoperative injection of anti-VEGF drugs before vitrectomy. In our study, PPV was completed during 3–7 days after intravitreal injection. Data indicated that drugs were effective and patient postoperative conditions were significantly improved. Furthermore, no significant development of proliferative lesions was observed in 6 m. Since the blood glucose level is one of the important factors that affect the development of PDR [52], in our study, all patients were asked to actively control blood glucose before and after surgeries, preventing hyperglycemia leading to surgical failure. Several studies reported that drugs caused the retinal pigment epithelium (RPE) tears [53, 54]. However, in our study, no RPE tears were found after intravitreal injection during follow-up. Since there are many factors in the formation of cataract, for example, silicone oil intraocular filling can also lead to cataract, our study did not include cataract as one of the surgical complication [7].

In conclusion, this study suggested that in a developing country such as China, PDR patients living in rural areas usually could not receive early and effective treatment due to inconvenient transportation and inadequate community health care services; therefore, it is essential to reduce the cost of surgical complications, reoperation, and long-term treatment. 23-G PPV surgery assisted by intravitreal injection of conbercept, ranibizumab, or TA for PDR had a significant impact on patient health condition and economic burden. The application of these drugs can reduce difficulty of the operation, improve the success rate of PPV surgery, and decrease the incidence of postoperative complications, therefore reducing the patient's economic burden in China. Conbercept and ranibizumab have equal effectiveness and achieved better results than TA. The safety and efficacy of the anti-VEGF drugs were confirmed in the treatment of complex PDR. However, our research is limited, as the observation time is short, the long-term effects and complications of drugs had not been well reflected. Function mechanism of these drugs is also not completely understood. In addition, the number of cases in this study is inadequate for a definitive conclusion. Therefore, these results also need to be proved by clinical trials of large sample sizes and extended follow-up period.

## Figures and Tables

**Table 1:** Baseline characteristics of participants with or without conbercept pretreatment.

	IVC (*n* = 20)	IVR (*n* = 19)	IVTA (*n* = 19)	*P* value
Sex				0.759
Male (eyes, %)	9 (11, 55%)	13 (13, 68.4%)	10 (11, 57.9%)	
Female (eyes, %)	8 (9, 45%)	6 (6, 31.6%)	5 (8, 42.1%)	
Age (yrs)				
Mean (SD)	60.74 ± 2.63	55.28 ± 5.16	57.49 ± 4.22	0.246
Type of diabetes (case, %)				0.527
1	3 (15.0)	4 (21.1)	2 (10.5)	
2	12 (6.0)	10 (52.6)	14 (73.7)	
Uncertain		5 (25.0)	5 (26.3)	3 (15.8)
Ocular profile (case, %)				
Study eye (left/right)	13/7 (65.0/35.0)	8/11 (42.1/57.9)	6/13 (31.6/68.4)	0.138
Previous history of laser	4 (20.0)	2 (10.5)	2 (10.5)	0.495
Lens status	3 (15.0)	4 (21.1)	2 (10.5)	0.663
Pathogeny (case, %)				
Nonclearing vitreous hemorrhage	9 (45.0)	9 (47.4)	8 (42.1)	0.914
Diffuse fibrovascular proliferation	4 (20.0)	3 (15.8)	5 (26.3)	0.125
Traction retinal detachment	7 (35.0)	7 (36.8)	6 (31.6)	0.573
Extent of vitreoretinal adhesion grade (case, %)				0.416
0	0 (0.0)	0 (0.0)	0 (0.0)	
1	2 (10.0)	4 (21.1)	5 (26.3)	
2	12 (60.0)	9 (47.4)	10 (52.6)	
3	6 (30.0)	6 (31.6)	4 (21.1)	
Duration of diabetes (y)				
Mean (SD)	24.25 ± 6.33	28.76 ± 5.27	25.98 ± 4.6	0.227
Mean BCVA (ETDRS letters)			0.531	
Mean (SD)	27.83 ± 6.78	25.31 ± 4.23	28.46 ± 7.55	
Snellen equivalent (range)	20/100–HM	20/100–20/2000	20/80–HM	
IOP (mmHg)				
Mean (SD)	15.24 ± 4.67	.64 ± 6.21	16.35 ± 2.89	0.395
Cardiovascular condition (case, %)	12 (60.0)	10 (52.6)	13 (68.4)	1.103
Hypertension (case, %)	15 (75.0)	11 (57.9)	14 (73.7)	0.587
Cerebral vascular disease (case, %)	5 (25.0)	7 (36.8)	4 (21.1)	0.862

**Table 2:** Baseline complexity surgery score of DR patients.

Surgery	IVC		IVR		IVTA	
	Cases	Complexity surgery	Cases	Complexity surgery	Cases	Complexity
	(*n* = 20)	Score	(*n* = 19)	Score	(*n* = 19)	Score
VH						
Absent (0)	11	0	10	0	11	0
Mild (+1)	2	2	3	3	2	2
Moderate (+2)	5	10	4	8	5	10
Severe (+3)	2	6	2	6	1	3
Amount of previous						
Retinal photocoagulation						
Complete PRP (0)	1	0	0	0	0	0
Incomplete PRP (+1)	2	2	1	1	2	2
Focal (+2)	1	2	1	2	0	0
None (+3)	16	48	17			1
Configuration of retinal detachment						
Absent (0)	13	0	12	0	13	0
Hammock (+1)	4	4	3	3	2	2
Central diffuse (+2)	3	6	4	8	4	8
Table-top (+3)	0	0	0	0	0	0
Total complexity surgery score	20	80	19	82	19	78
Means (SD)		4.00 ± 13.38		4.32 ± 14.23		4.11 ± 14.39
*P*		0.67 (IVC versus IVTA)		0.39 (IVR versus IVTA)		

**Table 3:** Primary outcomes (Mean ± SD).

	IVC	IVR	IVTA	*P* value^∗^
Mean BCVA improvement (ETDRS letters)				
(Mean ± SD)	25.10 ± 3.73	26.32 ± 4.06	17.16 ± 2.87	0.337, <0.01, <0.01
Operation time (minutes)				
(Mean ± SD)	56.65 ± 6.52	54.89 ± 6.46	77.32 ± 6.36	0.404, <0.01, <0.01
Incidence of iatrogenic retinal breaks (cases, %)	2 (10.0)	2 (10.5)	8 (42.1)	0.958, 0.024, 0.027
Endodiathermy rate (cases, %)	5 (25.0)	6 (31.6)	12 (63.2)	0.659, 0.014, 0.049
Silicone oil tamponade (cases, %)	9 (45.0)	9 (47.4)	15 (78.9)	0.885, 0.029, 0.045

^∗^ *P* value of IVC versus IVR, IVC versus IVTA, and IVR versus IVTA.

**Table 4:** Secondary outcomes and IOP.

	IVC	IVR	IVTA	*P* value^∗^
Vitreous clear-up time (days)				
(Mean ± SD)	6.10 ± 1.52	6.32 ± 1.57	11.11 ± 2.38	0.66, <0.01, <0.01
Intraoperative bleeding (cases, %)	2 (10.0)	3 (15.8)	9 (47.4)	0.602, 0.010, 0.04
Postoperative bleeding (cases, %)	1 (5.0)	1 (5.3)	3 (15.8)	0.971, 0.287, 0.305
PRP completion rate (cases, %)	11 (55.0)	10 (52.6)	6 (31.6)	0.886, 0.147, 0.199
Reoperation probability (cases, %)	1 (5.0)	1 (5.3)	2 (10.5)	0.971, 0.534, 0.560
IOP increase (case, %)	3 (15.0)	2 (10.5)	9 (47.4)	0.684, 0.031, 0.011

^∗^ *P* value of IVC versus IVR, IVC versus IVTA, and IVR versus IVTA.

**Table 5:** System adverse events compared with baseline.

IVC	IVR	IVTA	*P* value^∗^	
Cardiovascular disease (case, %)	14 (70.0)	13 (68.4)	14 (73.7)	0.519, 0.333, 0.729
Hypertension (case, %)	15 (75.0)	12 (63.2)	16 (84.2)	1.000, 0.748, 0.439
Cerebral vascular disease (case, %)	6 (30.0)	7 (36.8)	5 (26.3)	0.731, 1.000, 0.712

^∗^ *P* value of IVC, IVR, and IVTA.

## Supplementary Materials

Supplementary Materials Supplementary Table 1: detailed disease phenotypes of individual eye in three groups.
