# Supplementary material for: Efficacy and Safety of Intravitreal Conbercept, Ranibizumab, and Triamcinolone on 23-Gauge Vitrectomy for Patients with Proliferative Diabetic Retinopathy
Source: J Ophthalmol. 2018 Jun 25;2018:4927259. doi: 10.1155/2018/4927259 (PMC6036808; doi:10.1155/2018/4927259)
Supplement: Supplementary Materials — Supplementary Table 1: detailed disease phenotypes of individual eye in three groups. [file 4927259.f1.docx]

**Supplementary Table 1. Detailed disease phenotypes of Individual eye of three groups**

| **Eye #** | **VH** | | | **Amount of previous  Retinal Photocoagulation** | | | **Conﬁguration of retinal detachment** | | |
| --- | --- | --- | --- | --- | --- | --- | --- | --- | --- |
|  | **IVC** | **IVR** | **IVTA** | **IVC** | **IVR** | **IVTA** | **IVC** | **IVR** | **IVTA** |
| 1 | 2 | 0 | 0 | 3 | 3 | 3 | 1 | 0 | 0 |
| 2 | 0 | 2 | 0 | 3 | 3 | 3 | 0 | 2 | 0 |
| 3 | 0 | 1 | 2 | 3 | 3 | 3 | 0 | 0 | 2 |
| 4 | 1 | 0 | 0 | 3 | 3 | 1 | 0 | 0 | 0 |
| 5 | 2 | 2 | 1 | 3 | 3 | 3 | 1 | 1 | 0 |
| 6 | 3 | 0 | 2 | 3 | 3 | 3 | 2 | 0 | 2 |
| 7 | 0 | 3 | 0 | 3 | 3 | 3 | 0 | 2 | 0 |
| 8 | 0 | 0 | 0 | 0 | 3 | 1 | 0 | 0 | 0 |
| 9 | 1 | 2 | 0 | 3 | 3 | 3 | 0 | 1 | 0 |
| 10 | 3 | 0 | 1 | 3 | 3 | 3 | 2 | 0 | 0 |
| 11 | 0 | 0 | 2 | 3 | 3 | 3 | 0 | 0 | 1 |
| 12 | 0 | 0 | 0 | 1 | 2 | 3 | 0 | 0 | 0 |
| 13 | 0 | 1 | 0 | 3 | 3 | 3 | 0 | 0 | 0 |
| 14 | 2 | 0 | 2 | 3 | 3 | 3 | 2 | 0 | 2 |
| 15 | 0 | 2 | 3 | 3 | 3 | 3 | 0 | 2 | 1 |
| 16 | 0 | 0 | 0 | 1 | 1 | 3 | 0 | 0 | 0 |
| 17 | 2 | 1 | 2 | 3 | 3 | 3 | 1 | 1 | 2 |
| 18 | 0 | 0 | 0 | 2 | 3 | 3 | 0 | 0 | 0 |
| 19 | 2 | 3 | 0 | 3 | 3 | 3 | 1 | 2 | 0 |
| 20 | 0 |  |  | 3 |  |  | 0 |  |  |
| **Total** | **18** | **17** | **15** | **52** | **54** | **53** | **10** | **11** | **10** |
| **P Values** | **0.9883** | **0.452968** | **0.77199** | **0.297563** | **0.44403** | **0.789852** | **0.760166** | **0.919094** | **0.858462** |
| **Comparison pairs** | **P (IVC vs IVR)** | **P (IVC vs IVTA)** | **P (IVR vs IVTA)** | **P (IVC vs IVR)** | **P (IVC vs IVTA)** | **P (IVR vs IVTA)** | **P (IVC vs IVR)** | **P (IVC vs IVTA)** | **P (IVR vs IVTA)** |
